# The Voice Unheard: Women’s Perception of Maternal Health Care Post-Flint Water Crisis

**DOI:** 10.1007/s40615-025-02453-2

**Published:** 2025-05-20

**Authors:** Kionna L. Henderson, Ashton M. Shortridge, Richard C. Sadler, Caitlin Canfield, Alan L. Mendelsohn, Mahbuba Khan, Kent D. Key

**Affiliations:** 1 Baylor Scott & White Health and Wellness Center, Baylor Scott & White Health, Dallas, TX USA; 2 Department of Geography, Environment, and Spatial Sciences, Michigan State University, East Lansing, MI USA; 3 Department of Pediatrics, Division of Developmental-Behavioral Pediatrics, NYU Grossman School of Medicine, New York, NY USA; 4 Department of Public Health, College of Medicine, Charles Stewart Mott, Michigan State University, East Lansing, MI USA

**Keywords:** Maternal health disparities, Flint water crisis, Maternal morbidity, Healthcare discrimination, Maternal health effects

## Abstract

**Background:**

Eleven years have passed since the 2014 Flint water crisis (FWC), yet many voices still go unheard. There is limited evidence of the impact of the FWC on maternal health. This paper used a cross-sectional study design to survey 152 women enrolled in the Supporting Parents and Raising Resilient Kids (SPARRK) study in Flint, Michigan to examine racial differences in women’s perceptions of their overall health pre- and post-FWC, perceived maternal health services, and explore the interaction of race and living in Flint on maternal morbidity.

**Methods:**

Perceived maternal health was defined using the Centers for Disease Control and Prevention’s 21 Severe Maternal Morbidity (SMM) diagnosis codes. SMM were obtained via questionnaire. Logistic regression analyses were performed to identify factors associated with SMM within two domains: (1) overall health pre- and post-FWC and (2) perceived maternal health care received during birth.

**Results:**

There were 17 cases of SMM in which Black women accounted for 62.5% of these cases. Perceived quality of care was overall positive; yet, perceived overall health decreased post-FWC for all women. The odds of SMM were 6 times higher for those who had a college degree or higher.

**Conclusion:**

In the predominately Black city of Flint, race was not a significant factor in the perception of health and quality of care. Surprisingly, educational attainment was significantly associated with a 6-time increase in odds of experiencing an SMM. More research is needed to examine the association of patient-provider perception of quality care and education on maternal health outcomes.

## Introduction

Eleven years have passed since the public outcry of the Flint water crisis (FWC), yet many voices remain unheard. The Michigan Civil Rights Commission conducted an investigation which included resident hearings to gather experiences of the water crisis. From these findings, the commission issued a report to Flint residents declaring the FWC and empathizing with their suffering—often referencing the lack of trust in the government and decades of systemic racism and discrimination [1]. Despite the public’s responsiveness to the FWC, there remains a demand for action and strategic plans to support and care for the needs of Flint residents, the plurality of whom are Black [2]. Studies on the FWC have investigated the effects of lead ingestion on child development, but no research has examined residents’ perceptions of the impact of the water crisis on maternal health [3–6].

Exposure to an environmental hazard such as drinking water contamination can increase overall maternal morbidity [7, 8]. Nationwide, Black women are 3–4 times more likely to be diagnosed with a SMM or die during childbirth compared to White women [9]. A Black woman’s exposure to this type of environmental hazard nearly doubles their risk of severe maternal morbidity (SMM). Many people of color are more likely to encounter chronic stressors such as daily exposure to individual and systemic discrimination (e.g., weathering effect) which can add to an increased the risk of adverse health outcomes [7, 8, 10, 11]. Scholars have also found that structural discrimination and inequities contribute to an increase in the risk of SMM and/or maternal death [12, 13]. A 2020 report examining the maternal health status of 38 states found that 30% of maternal deaths were attributed to discrimination [14]. Another report on racial differences in the healthcare system found that 21% of Black women have reported being treated unfairly due to their race by hospital personnel [15]. Race, especially identifying as African American/Black, has been shown to be an influential factor driving stark differences in maternal health outcomes and the quality of services received from healthcare systems. Moreover, in the context of residential segregation, which has a long history within the city of Flint, Black and Hispanic women may have higher odds of SMM [16]. While there are programs to address lead consumption and Flint’s economic distress [17], there are limited efforts to examine and provide resources for maternal health outcomes post-FWC [4, 18].

We sought to address this gap by examining SMM and perception of maternal health experiences in Flint following the FWC. The objectives of this study are to examine racial differences in women’s perceptions of their health pre- and post-FWC, explore the presence of maternal health service discrimination among women who delivered a child in Flint post-FWC, and identify the association of race by living in Flint on maternal health—defined as the experience of a severe maternal morbidity. We address three main research questions (RQ): (1) *To what extent do women in Flint perceive their overall health changed after the FWC, and do those perceptions differ by race?* (2) *What have women’s pregnancy experiences with the health care system been, how do women perceive their maternal health care experiences post-FWC, including experiencing discrimination, and what is the association between perception of maternal health care experiences and SMM?* and (3) *To what extent does the interaction of race and residing in the city of Flint, a city burdened by racial segregation, have on SMM*? *What is the association of perceived health care plus this interaction term on SMM?*

To our knowledge, this is the first study to investigate the perceptions of maternal health for women who delivered in Flint post-FWC. Health can be defined both subjectively and objectively, with perception of health and health care playing a vital role in physical health status [19–21]. We hypothesize negative perceptions and experiences of maternal health care after the FWC to be more prevalent among Black women compared to White women in Flint.

## Conceptual Framework

**Fig. 1:**
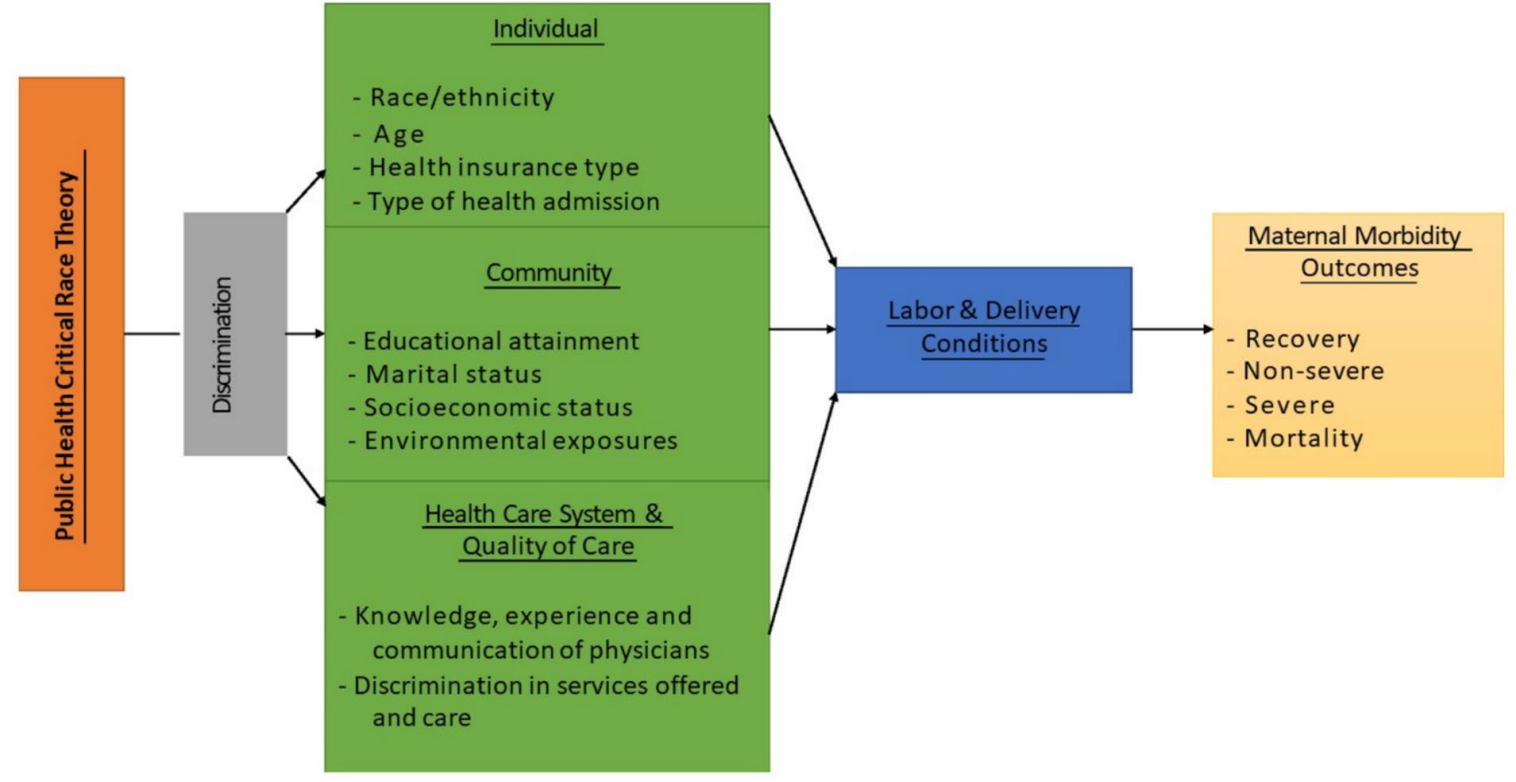
Modified pathways of institutional discrimination on maternal health disparities

Our conceptual framework includes a modified version of the Pathways of Institutional Discrimination on Maternal Health Disparities [22] (Fig. 1). The original framework integrates three concepts: (1) Public Health Critical Race Theory (PHCRT), (2) the World Health Organization maternal morbidity framework, and (3) the maternal morbidity concept [23–25]. The framework proposes that racial discrimination acts across both sociodemographic characteristics and geographic scales (i.e., individual and community) to influence adverse labor and delivery outcomes within the healthcare system. The modified framework includes environmental exposures, such as the Flint water crisis, as a factor at the community level.

## Methods

We performed a secondary analysis utilizing retrospective data collected during baseline assessments in an ongoing randomized control trial (RCT), the *Supporting Parents and Raising Resilient Kids (SPARRK)* study. The larger study’s overall goal was to determine the role of parenting support—via PlayReadVIP (formerly known as Video Interaction Project)—on preventing adverse outcomes among young children in the aftermath of the FWC [26]. The SPARRK study was registered with clinicaltrials.gov (NCT03945552), and IRB approval was obtained with the NYU Grossman School of Medicine IRB acting as the single IRB for the study (#s18-01347).

Inclusion criteria for child participants were 32-week gestational age or greater, birthweight of 1500 g or greater, singleton birth, no significant neonatal or medical complications, and no known or suspected significant genetic abnormalities, neurodevelopmental disorders, neuromuscular conditions, or visual/hearing impairments. For caregivers, inclusion criteria were English speaking, had custody of child, no significant communication impairment, and planned to continue receiving pediatric care at the Hurley Children’s Clinic. Families were enrolled within three months of their first in-person pediatric visit.

A total of 486 women voluntarily consented for themselves and their infants to participate in the SPARRK study. Four hundred eight mothers were randomized to either the PlayReadVIP or Control groups and completed baseline assessments, which included questions regarding demographic characteristics and standardized measures of mental health, material hardship, and social support. For this study, we surveyed a subset of participants (*n* = 152) about perceptions of overall health, prenatal health care, and experience of SMM. Data from mothers who identified as White or Black (*n* = 140) were used in the present analyses. Residential association and SMM were explored for mothers who live in the city of Flint compared to those who do not.

### Measures

We assessed perceptions on two themes: (1) overall health pre-FWC compared to post-FWC and (2) maternal health care experiences post-FWC—specially examining the context of perceived discrimination among health care providers. Survey questions for these domains were adapted from Pregnancy Risk Assessment Monitoring System (PRAMS) questionnaire [27].

#### Overall Health and FWC Experience

The participants answered a series of questions related to their overall health pre- and post-FWC*.* Participants were asked “How would you rate your overall health before the Flint Water Crisis (before 2014)?” compared to their health “How would you rate your overall health after the water system was switched in Flint (2014–present)?” To gauge the current presence of comorbidities, participants were asked if they had the following health conditions since their child was born: high blood pressure, type 1 or type 2 diabetes, depression, and/or gestational diabetes.

#### Maternal Health Care and SMM Experiences

Additional questions were asked about perceptions of their health care experiences during pregnancy. The respondent rankings for perceived health questions were as follows: “Never,” “Often,” “Sometimes,” “Rarely,” “Unsure,” and “No answer.” The outcome variables concerned the following themes: (1) perceived quality of maternal health care received post-FWC in regard to be feeling listened to, treated with respect, and receiving quality health care services from hospital personnel (*e.g., … did you feel like the physicians, nurses and/or hospital personnel listened to you or treated you with less respect or differently due to your race, culture, ethnicity, or age?)* and (2) experience of SMM (*e.g., … has a doctor, nurse, or other health care professional ever told you that you had one of the following severe maternal morbidity conditions?)*. The Centers for Disease Control and Prevention (CDC) defines SMM using 21 indicators [28]. For this study, we selected common SMM indicators, adapted the diagnosis descriptions to enhance participant understanding, and provided the updated list of diagnoses to the study participants. Although this measure relied on participant-report only, prior research has indicated that maternal recall of prenatal and perinatal health adequately agrees with medical records [29].

Table 1 under the “Results” section includes this list as well as the percentage of each SMM case by race.

### Residing in the City of Flint

Residential addresses at the time of birth were obtained for all participants of the SPARRK study. We joined these addresses to the census tract (proxy for neighborhood) using the 2020 American Community Survey (ACS) – US Bureau of Census. Our goal was to account for the proximity of participants who lived within Flint city limits compared to those who do not.

#### Sociodemographic

Demographic data were obtained from SPARRK baseline surveys which included the following variables: race (*Black, White, or Other*), ethnicity (*Hispanic or Non-Hispanic*), marital status (*single, married, or separated*), education (*less than a 12*^*th*^* grade education, some college including associate degree, college degree considered as bachelor’s degree, and professional degree*), income (*estimated income*), employment (*currently employed or not currently employed*), Medicaid (*served as health insurance indicator*), and pre-pregnancy comorbidities (*high blood pressure, hypertension, depression, gestational diabetes, Type 2- diabetes).* All of these factors have been shown to be associated with severe maternal morbidity (SMM) [24]. For our analysis, the ethnicity variable was removed due to the low number of respondents. Table 1 in the “Results” section provides a summary of demographic variables.

### Statistical Analyses

Descriptive statistics were calculated using RStudio to provide summary data of the study population (e.g., total number of participants, race categories, SMM counts) [30]. The following subsections describe the statistical methodology used to address each of the research questions:

- *RQ1:* Our first aim was to measure racial differences in women’s perception of their overall differences in health status after the FWC. To address this aim, we used contingency tables. The changes in health were then estimated by calculating the difference in responses (Table 3).
- *RQ2:* The second aim focused on evaluating women’s prenatal health experiences, how women perceive their maternal health care experiences post-FWC and experiences of discrimination, and what is the association between perceived maternal health care experiences on SMM. Observable differences in women’s perceived experiences were graphed. Separate logistic regression analyses were performed to examine factors associated with perceived health care quality (Model I) and SMM experience. We examined whether experience of SMM was associated with perceived health care quality (Model II). First, we conducted an analysis to determine if sociodemographic variables, including race (*Black or White*), marital status (*single, married, or separated*), education level (*less than high school diploma or high school diploma, some college, college degree, or professional degree*), income (*counted as a continuous variable*), employment status (*job or no job*), and presence of Medicaid (*served as a health insurance indicator*) predicted SMM and perceived health care quality

$$Model I:perceived\;health\;care\;quality=race+marital\;status+education+income+employment+Medicaid$$

$$Model II:SMM=perceived\;health\;care\;quality+race+marital status+education+income+employment+Medicaid$$

- *RQ3:* The final aim was to (1) determine to what extent is the interaction of race and residing in the city of Flint (interaction term) associated with severe maternal morbidity and (2) examine the association of the perceived health care plus the interaction term on SMM. To measure this aim, we geocoded the addresses of all women in the study sample using the Google Maps API through R’s ggmap library [31]. Incomplete addresses and non-Michigan addresses were excluded due to uncertainty. These records were spatially joined with their associated census tracts with IPUMS attribute data [32]. Using QGIS [33], a binary field was calculated (“1” if a participant lives within Flint census tracts; “0” if not). We then repeated the models from RQ2 including living in Flint as an independent predictor. Finally, we conducted logistic regression with interaction terms for both Black and White women who lived in Flint (e.g., race = “Black” * Flint census tract = 1) to identify if there was a race-specific association with living being in the city of Flint.

$$Model III:SMM=race+marital status+education+income+employment+Medicaid+race*lives in Flint$$

$$Model IV:SMM=perceived\;health\;care\;quality+race+marital status+education+income+employment+Medicaid+race*lives in Flint$$

## Results

Overall, two thirds of mothers identified as Black (*n* = 85). Most mothers received Medicaid (80.7%), and nearly 60% were employed. Most mothers were not married, and approximately half had a high school education or less. Among participants who identified as Black, there were 9 SMM cases. All Black participants identified as Non-Hispanic, and the majority were insured with Medicaid (87.1%) and employed (62.5%). Additionally, a substantial number of Black participants identified their marital status as “Single” (96.5%), and the highest educational attainment was a “High school diploma or less” (55.3%). Among White participants in our sample (*n* = 55), there were 8 SMM cases. Most White participants identified as Non-Hispanic (96.4%) and were insured by Medicaid (70.9%). A little over half of the White participants stated that they were employed (52.6%). Similar to Black participants, the majority of White participants identified as “Single” (72.7%). The highest educational attainment for White women was between “High school diploma or less” and “Some college.” Overall, household income averaged at $38,471.7. White participants had a higher mean household income ($57,137) compared to Black participants ($25,032.6) although more Black participants were employed.

**Table 1:** Summary table of study participants’ demographics

Variable	All	Black	White
*N*	140	85	55
Total SMM case, *n* (%)	17 (9.29)	9 (9.4)	8 (14.5)
Unique number of participants who reported SMM, *n* (%)	13 (9.3%)	8 (9.4)	5 (9.1)
Ethnicity, *n* (%)			
Hispanic	2 (1.4)	-	2 (3.6)
Non-Hispanic	138 (98.6)	85 (100.0)	53 (96.4)
Medicaid, *n* (%)			
Yes	113 (80.7)	74 (87.1)	39 (70.9)
No	27 (19.3)	11 (12.9)	16 (29.1)
Employment status^a^, *n* (%)			
Employed	40 (59.7)	30 (62.5)	10 (52.6)
Not employed	27 (40.3)	18 (37.5)	9 (47.4)
Yearly household income^b^, mean (SD)	38,471.7 (58,416.7)	25,032.6 (20,395.6)	57,137.0 (540,000.0)
Marital status, *n* (%)			
Single or separated	122 (87.1)	82 (96.5)	40 (72.7)
Married	18 (12.9)	3 (3.5)	15 (27.3)
Education			
High school diploma or less	70 (50.0)	47 (55.3)	23 (41.8)
Some college	52 (37.1)	30 (35.3)	22 (40.0)
College degree or higher than college degree or professional degree	18 (12.9)	8 (9.4)	10 (18.2)

^a^Sixty-seven participants (48 Black and 19 White) had data for employment status. ^b^One hundred twenty-nine participants (75 Black and 54 White) reported yearly household income

There were 17 overall cases of SMM between Black and White women in the analytic sample. Among the Black SMM cases, blood clots were the most frequent, with Black women accounting for 62.5% of these cases (Table 2). More Black women in our sample also experienced blood transfusions than White women. Within White SMM cases, White women had more experiences in heart failure, sepsis, eclampsia, and ventilation than Black women.

**Table 2:** SMM indicators, adjusted term adapted by survey team, and total SMM cases by race

SMM indicator term	Adapted survey term	SMM cases in Black, *n* (%)	SMM cases in White, *n* (%)	Total SMM cases, *n* (%)
		Total number of SMM in Black = 9	Total number of SMM in White = 8	Total number of SMM = 17
Acute renal failure	Kidney failure	0	0	0
Adult respiratory distress syndrome	Fluid in lungs	0	0	0
Cardiac arrest, fibrillation, or conversion of cardiac rhythm	Heart failure/stroke/heart arrest	0	1 (12.5)	1 (5.8)
Shock	Shock	0	0	0
Acute myocardial infraction or aneurysm	Heart attack	0	0	0
Sepsis	Severe bacterial infections during or shortly after pregnancy	0	1 (12.5)	1 (5.8)
Hysterectomy	Removal of cervix	0	0	0
Disseminated intravascular coagulation	Blood clots/abnormal bleeding	5 (55.6)	3 (37.5)	8 (47.1)
Acute congestive heart failure	Heart failure/fluid around heart	0	0	0
Eclampsia	Seizures/coma/toxic pregnancy	1 (11.1)	2 (25.0)	3 (17.6)
Blood transfusion	Blood transfusion	3 (33.3)	0	3 (17.6)
Ventilation/temporary tracheostomy	Ventilation/temporary tracheostomy to assist with breathing	0	1 (12.5)	1 (5.8)

### RQ 1: Perceived Health Overall Pre- and Post-FWC

Overall, perceived health post-FWC ratings decreased for women. Comparing the differences in overall health pre-FWC vs post-FWC period, 79% of Black women and 81% of White women rated their health as less than “Excellent” post-FWC. In the post-FWC period, we saw that 25% more Black women and 10% more White women rated their health as less than “Excellent.” The most notable differences in ratings in overall health were observed in the “Excellent” and “Fair” health ratings for all women. Table 3 shows the difference in overall health ratings among participants pre- vs post-FWC.

**Table 3:** Difference in overall health rating pre-FWC compared to post-FWC

Health ratings	Pre-FWC		Post-FWC	
	Black	White	Black	White
Excellent	41 (45%)	15 (29%)	18 (20%)	10 (19%)
Good	36 (39%)	28 (54%)	40 (43%)	30 (58%)
Fair	13 (14%)	7 (13%)	25 (27%)	9 (17%)
Poor	2 (2%)	1 (2%)	8 (9%)	2 (4%)
No answer	0	1 (2%)	1 (1%)	1 (2%)

### RQ 2: Perceived Prenatal Health Experiences and Its Association with SMM

**Fig. 2:**
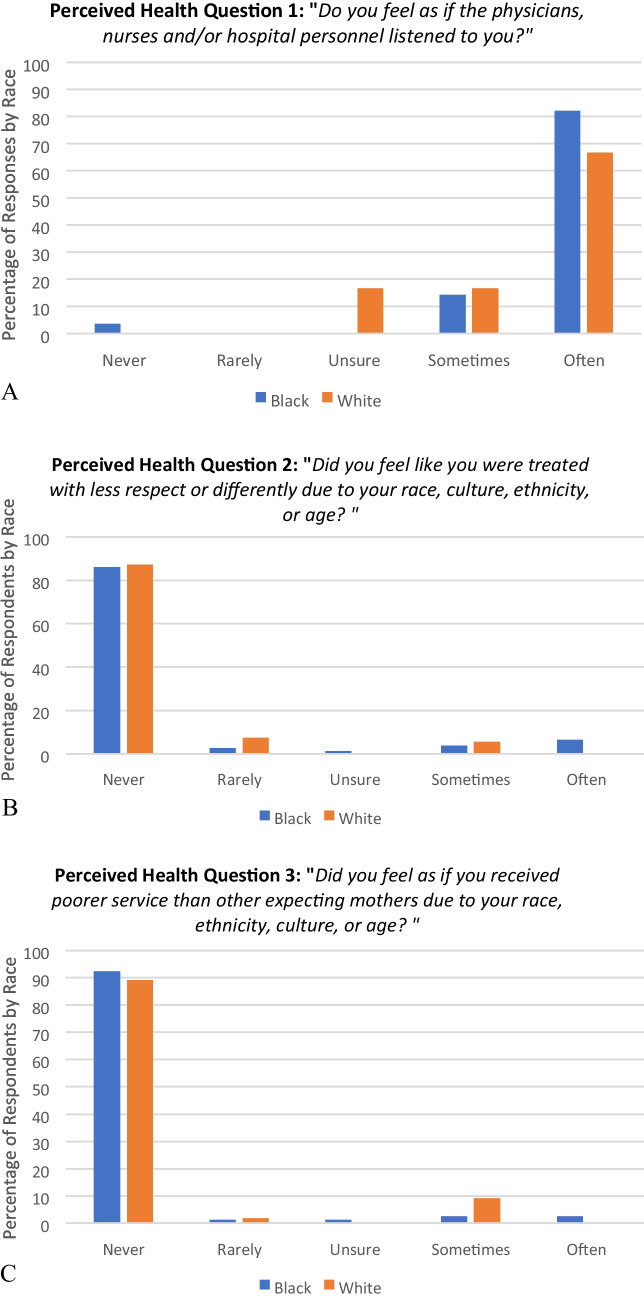
**A**–**C** Perceived health care services questionnaire summary

Regardless of race, a majority of respondents had positive experiences in regard to being listened to and respected and receiving quality services during childbirth. Among the perceived health care questions that referred to women being asked if they felt *listened to* about their feelings of pain and discomfort during childbirth (Figure 2A), majority of participants felt that they were “Often” listened to. Nearly 4% of Black respondents perceived they were “Never” listened to by hospital personnel, and 80% responded that they were “Often” listened to. Participants were asked if they were *treated with less respect* or differently due to [their] race, culture, ethnicity, or age (Figure 2B). Of the respondents, majority responded that they “Never” felt this way. Furthermore, when asked about the quality of services during childbirth (Figure 2C), over half of the participants stated that they “Never” received poorer services due to their race, culture, ethnicity, or age. More White women than Black women stated that they “Sometimes” received lower quality services, and more Black women than White women responded that they “Often” received poorer services.

We examined whether perceived health (PH) was associated with individual sociodemographic factors using logistic regression analysis and controlling for participant factors (Model I). Employment status was removed due to the number of missing values and the inability to determine significance. No significant association was observed for PH Questions 1, 2, or 3 (*results not shown*). When determining the association of SMM and perceived health (Model II), Employment and Perceived Health Question 1 were excluded from the analysis due to missing values. We found that the odds of SMM were over 6 times significantly higher for those who had a “College degree or higher than college degree or professional degree” for PH Questions 2 and 3 (Tables 4 and 5). For every $1000 dollar increase in annual household income, the odds of SMM among women significantly decrease by 4.6% or 0.954 times lower, keeping all other variables constant in the model for both perceived health questions. Race, marital status, education attainment level (*High school diploma or less*), household income, and Medicaid were not significantly associated with the odds of SMM among the survey participants.

**Table 4:** Estimates of odds ratio and 95% confidence interval for predictors of SMM, including sociodemographic factors and PH Question 2 (“being treated with respect”)

Variable	Odds ratio	95% confidence interval	*P*-value
Perceived Health Q2: (birth respect)			
Answered “Never”	0.753	(0.197, 2.882)	0.6788
Answered other categories (ref)	-	-	-
Race			
Black	0.802	(0.214, 3.004)	0.7435
White (ref)	-	-	-
Marital status			
Single or separated	0.727	(0.099, 5.353)	0.754
Married (ref)	-	-	-
Education			
High school diploma or less	0.485	(0.109, 2.149)	0.3406
College degree or higher than college degree or professional degree	6.404	(1.111, 36.931)	0.0378*
Some college (ref)	-	-	-
Annual household income in thousand	0.954	(0.911, 0.999)	0.0465*
Medicaid			
Yes	0.943	(0.166, 5.353)	0.9468
No (ref)	-	-	-

*Significant at *p*-value of 0.05

**Table 5:** Estimates of odds ratio and 95% confidence interval for predictors of SMM, including sociodemographic factors and PH Question 3 (“receiving poorer birthing services”)

Variable	Odds ratio	95% confidence interval	*P*-value
Perceived Health Q3: (birth service)			
Answered “Never”	0.894	(0.204, 3.923)	0.8818
Answered other categories (ref)	-	-	-
Race			
Black	0.816	(0.217, 3.063)	0.7631
White (ref)	-	-	-
Marital status			
Single or separated	0.697	(0.096, 5.042)	0.7209
Married (ref)	-	-	-
Education			
High school diploma or less	0.493	(0.111, 2.199)	0.354
College degree or higher than college degree or professional degree	6.557	(1.127, 38.162)	0.0364*
Some college (ref)	-	-	-
Annual household income in thousand	0.954	(0.911, 0.999)	0.0457*
Medicaid			
Yes	0.937	(0.163, 5.387)	0.9414
No (ref)	-	-	-

*Significant at *p*-value of 0.05

### RQ 3: Association of Race by Residing in Flint on SMM

We estimated the association between SMM and the interaction of race by living in the city of Flint using logistic regression (Model III). There were no significant variables associated with Model III. Results for this analysis are not shown. In Table 6, we examined Model IV which includes the association between SMM, perceived health care, and the interaction term (race * living in Flint). We found that similar to previous analysis, Employment and Perceived Health Question 1 were excluded due to missing values. Similarly, to Model II, we found that Educational attainment and Annual income were both significantly associated with an increased odds of SMM when controlling for the interaction term. Participants with a College degree or higher than a college degree or professional degree had a 6-time increase in the odds of experiencing a SMM compared to participants with Some college education. PH Questions 2 and 3, race, marital status, Medicaid, and living in Flint were not significant.

**Table 6:** Estimates of odds ratio and 95% confidence interval for predictors of SMM, including socioeconomic factors, PH Question 2, and living in flint * race

Variable	Odds ratio	95% confidence interval	*P*-value
Perceived Health Q2: (birth respect)			
Answered “Never”	0.752	(0.188, 3)	0.6863
Answered other categories (ref)	-	-	-
Race			
Black	0.802	(0.214, 3.005)	0.7437
White (ref)	-	-	-
Marital status			
Single or separated	0.726	(0.097, 5.405)	0.7545
Married (ref)	-	-	-
Education			
High school diploma or less	0.485	(0.109, 2.15)	0.3407
College degree or higher than college degree or professional degree	6.398	(1.099, 37.269)	0.039*
Some college (ref)	-	-	-
Annual household income in thousands	0.954	(0.911, 0.999)	0.047*
Medicaid			
Yes	0.943	(0.165, 5.391)	0.9476
No (ref)	-	-	-
Flint			
Lived within Flint	1.006	(0.285, 3.552)	0.9926
Did not live within Flint (ref)	-	-	-

*Significant at *p*-value of 0.05

## Discussion

This study set out to explore the extent of racial differences in women’s perceptions of maternal health post-FWC within two categories: (*1) overall health changed after the FWC, and (2) maternal health care experiences post-FWC, including whether they experienced discrimination.* Our team also determined to what extent residing in the city of Flint, a city burdened by racial segregation, is associated with both maternal morbidity and perceived health care quality among Black and White women. Within our sample, we found that race was not a driving factor in increased rates of maternal morbidity in Flint. Among the perceived health care quality variables related to feeling respected and services provided, we found that educational attainment of a college degree or higher and annual income was significantly associated with an increase chance of SMM. This finding is in alignment with a recent report by the Morbidity and Mortality Weekly Report that found that Black, college educated women are 1.6 times more likely to experience maternal death compared to White women of lesser educational status [34].

While our study examined the differences in perceived health pre- and post-FWC, we did not collect biological maternal evidence or vital statistic records pre-, during, and post-FWC to determine if and to what extent the environmental effects of the water crisis contributed directly to any of the maternal results observed. Our study was able to capture a snapshot of the perceived health before the water crisis and compared it to health post-FWC. Furthermore, we were able to examine the current perceived state of maternal health after an environmental hazard. To our knowledge, this has not been examined in the current literature on the FWC. The impact of environmental hazards on maternal health outcomes has shown to negatively impact factors such as chronic health diseases and fertility challenges [35]. A study by Grossman and Slusky in 2019 found that fertility decreased by 12% post-FWC and overall health declined [4]. This finding supports our study’s results as overall perceived health declines post-FWC for both Black and White women.

Our conceptual framework, a modification of the *Pathways of Institutional Discrimination on Maternal Disparities*, suggests that Black women would have higher rates of SMM due to systemic and institutional opposing factors. Nationwide, Black women typically have carried the burden of SMM; however, our study found that within the predominately Black city of Flint, maternal health outcomes do not significantly differ across race. Our findings differ from other same-site delivery studies such as that of Howell et al. in 2016, who found that, in New York City, women who delivered a child at similar health systems had different maternal health outcomes by race [36]. In California, Mujahid et al. found that birthing hospitals with high ranking SMM rates accounted for only 8% of the maternal morbidity differences between Black and White women [37]. The California study suggested that root causes within institutions and their association with SMM should be reviewed. Within our study, we found that despite residing in a predominantly Black city—that has been disenfranchised and institutionally oppressed for decades—race was not significantly associated with severe maternal morbidity (Tables 6 and 7).

**Table 7:** Estimates of odds ratio and 95% confidence interval for predictors of SMM, including socioeconomic factors, PH Question 3, and living in flint * race

Variable	Odds ratio	95% confidence interval	*P*-value
Perceived Health Q3: (birth service)			
Answered “Never”	0.902	(0.2, 4.071)	0.8933
Answered other categories (ref)	-	-	-
Race			
Black	0.815	(0.217, 3.06)	0.7615
White (ref)	-	-	-
Marital status			
Single or separated	0.703	(0.096, 5.151)	0.7285
Married (ref)	-	-	-
Education			
High school diploma or less	0.494	(0.111, 2.206)	0.3558
College degree or higher than college degree or professional degree	6.601	(1.12, 38.896)	0.037*
Some college (ref)	-	-	-
Annual household income in thousand	0.954	(0.911, 0.999)	0.047*
Medicaid			
Yes	0.933	(0.162, 5.378)	0.9378
No (ref)	-	-	-
Flint			
Lived within Flint	0.96	(0.276, 3.335)	0.9486
Did not live within Flint (ref)	-	-	-

*Significant at *p*-value of 0.05

However, our study provides an incentive for future research studies to further examine the observed overall perceived health decrease post-FWC for all women. This finding addresses our first research question (i.e., *Do women in Flint perceive their overall health improved after the FWC?*). Surprisingly, we found that educational attainment, specifically having a college degree or higher, not race significantly increased of odds of SMM by 6 times. While this is an unexpected outcome in our study, this finding supports our conceptual model as educational attainment is among the factors impacted by discrimination that potentially contribute to differences in labor and delivery treatments resulting in maternal health outcome disparities—especially among Black women. It is important to note that due to our small sample size, it is likely that the number of mothers within this category contributed to the substantial rate. Nonetheless, while difficult, more research on educational attainment by race and a larger sample size conducted within the healthcare system (e.g., before, during, and after the birthing experience) are needed to study racial differences in quality of care throughout pregnancy and after birth. We recommend that more research at the community level with a larger number of participants is needed to better solidify this finding.

Study participants reported similar maternal health care quality experiences, addressing our second research question on perceptions of maternal health services. Most women regardless of race reported satisfaction with the quality of treatment and respect that they experienced. A recent study found that race concordance among birthing providers and patients significantly improved birth outcomes among Black patients; yet its impact on maternal health outcomes was not substantial [38]. More research with a larger sample size that includes prospective perception of maternal health quality is needed to examine the effect of SMM and perceived quality of care by race. Lister et al. (2019) align with our sentiments on the importance of perception as they found that the Black women in their focus groups reported experiencing racism and bias from providers due to their insurance type and receiving lower quality of prenatal services [9]. The voices of women, specifically Black women, may go unheard due to the rare events of maternal morbidity and mortality. However, when death is an extreme adverse outcome of labor and delivery, these rare cases should not go unheard.

Similarly, due to the demographic and socioeconomic makeup of Flint, we expected Black women to have negative perceptions of health care services received. Assessing the perceived health care services received post-FWC, we observed that the majority of the views of discrimination in services provided are positive with less discrimination. While not significant, a small percentage of Black women in our sample perceived that they were given differential treatment in services provided and respect and were being listened to during childbirth (Figure 2B). More information is needed on the demographic, economic, and board certification of the birthing providers to determine if which component positively affected women’s birthing experience. Additionally, we recommend further research identifying the association of providers who are impacted by workplace discriminatory policies on patient health. Serafini et al. identified how perceived challenges and enablers among racial and ethnic providers within health systems including discriminatory practices and lack of support substantially impact the well-being of racial and ethnic providers [39].

Based on our findings, we encourage healthcare system policies to train all hospital personnel in cultural competency with a focus on reducing explicit/implicit biases towards racial differences and educational attainment status. We also encourage quality assessment interventions to ensure all women are provided with quality care, listened to, and respected within the healthcare system regardless of race, age, gender, and/or cultural backgrounds. An example of a maternal health disparity intervention that could be tailored to the healthcare system within the context of reducing racial and educational attainment discrimination is the windshield tours of the Racial and Ethnic Approaches to Community Health (REACH) initiative [40]. The goal of this project was to utilize community-based efforts to reduce maternal and child health disparities. The intervention allowed healthcare system staff, who typically did not reside in the city, to tour the neighborhoods of the patients to become aware of the challenges many faced in accessing care. Robust healthcare system-level policies and/or interventions to address maternal health outcomes and quality of care are needed to reduce long-standing maternal health disparities.

## Limitations

Our study was designed using a retrospective, cross-sectional framework which limits the ability to determine causality. However, the analyses conducted contribute to the overall literature on the city of Flint and maternal health. Using self-reported SMM to define maternal health outcomes could potentially incorporate recall biases within our sample. Nevertheless, self-reported experiences have been demonstrated to be an effective and commonly used method for data collection within the health care field [41–43]. Another limitation is the inability to generalize the study results to other cities as Flint might be a special case due to it being a post-war industrial city, having gone through years of economic decline, and then experiencing the shock of the FWC. Though unique, the findings here create a foundation for future studies to examine environmental hazards involving drinking water’s impact on maternal health. Additionally, not incorporating qualitative measures such as focus groups or informational interviews to fully understand health quality–related issues among participants. Lastly, with the available data, there is no way to determine if the participant lived in the same residence during the FWC as when they were surveyed in 2022.

## Conclusion

This study found that race was not significantly associated with SMM rates in Flint post-FWC; however, having a college degree or equivalent was substantially associated with higher odds of an adverse maternal health outcome. Examining the maternal health perceptions of women who delivered in Flint, the overall perceived health has declined post-FWC for all women. More research on this specific topic and a larger sample size is needed to confirm differences in perceived maternal health care services.

The purpose of this study was to shine a light on the issues within healthcare systems in predominately Black cities and possibly evoke healthcare system policy change among hospital directors, staff, and key personnel to create intervention strategies that promote maternal health equity among pregnant women. Health professionals should take on the challenge of identifying disproportionalities in the healthcare system and the surrounding environment. Every woman regardless of race, age, socioeconomic status, address, and education should have the right to have an equitable, successful birth and a quality birthing environment. It is our hope that this research will provoke conversations among healthcare system policy makers to understand and work towards eliminating negative maternal health outcomes associated with harmful environmental exposures and addressing systemic racism in the healthcare field.

## Data Availability

No applicable as the SPARRK study has to maintain the privacy and confidentiality of its participants.

## References

[1] “The Flint water crisis: systemic racism through the lens of flint, Report of the Michigan Civil Rights Commission,” Feb. 2017. [Online]. Available: https://www.michigan.gov/-/media/Project/Websites/mdcr/mcrc/reports/2017/flint-crisis-report-edited.pdf?rev=4601519b3af345cfb9d468ae6ece9141. Accessed 11 Nov 2024.

[2] “Flint, MI,” DataUSA. [Online]. Available: https://datausa.io/profile/geo/flint-mi/. Accessed 13 Nov 2024.

[3] Danagoulian S, Jenkins D. “Deadly delays maternal mortality in Peru: a rights-based approach to safe motherhood.” Washington, D.C.; 2007. [Online]. Available: https://phr.org/wp-content/uploads/2007/11/maternal-mortality-in-peru2007.pdf. Accessed 11 Nov 2024.

[4] Grossman DS, Slusky DJG. The impact of the Flint water crisis on fertility. Demography. 2019;56(6):2005–31. 10.1007/s13524-019-00831-0. 31808102 10.1007/s13524-019-00831-0

[5] Hanna-Attisha M, LaChance J, Sadler RC, Champney S. Elevated blood lead levels in children associated with the Flint drinking water crisis: a spatial analysis of risk and public health response. Am J Public Health. 2016;106(2):283–90. 26691115 10.2105/AJPH.2015.303003 PMC4985856

[6] Sadler RC, Highsmith AR. Rethinking Tiebout: the contribution of political fragmentation and racial/economic segregation to the Flint water crisis. Environ Justice. 2016;9(5):143–51.

[7] Morello-Frosch R, Shenassa ED. The environmental ‘riskscape’ and social inequality: implicationsfor explaining maternal and child health disparities. Environ Health Perspect. 2006;114(8):1150–3. 10.1289/ehp.8930. 16882517 10.1289/ehp.8930 PMC1551987

[8] Institute of Medicine (US) Committee on Environmental Justice. “Toward environmental justice: research, education, and health policy needs,” Washington, D.C.: National Academics Press (US); 1999. 23035313

[9] Lister RL. Black maternal mortality-the elephant in the room. World J Gynecol Womens Health. 2019;3(1):10–33552. 10.33552/WJGWH.2019.03.000555. 10.33552/wjgwh.2019.03.000555 PMC7384760 32719828

[10] Geronimus AT. The weathering hypothesis and the health of African-American women and infants: evidence and speculations. Ethn Dis. 1992;2(3):207–21. 1467758

[11] White TM, Borrell LN, El-Mohandes A. A review of the public health literature examining the roles of socioeconomic status and race/ethnicity on health outcomes in the United States. J Racial Ethn Health Disparities. 2024. 10.1007/s40615-024-02195-7. 39468002 10.1007/s40615-024-02195-7

[12] van Daalen KR, et al. Racial discrimination and adverse pregnancy outcomes: a systematic review and meta-analysis. BMJ Glob Health. 2022;7(8):e009227. 10.1136/bmjgh-2022-009227. 35918071 10.1136/bmjgh-2022-009227 PMC9344988

[13] Hailu EM, Maddali SR, Snowden JM, Carmichael SL, Mujahid MS. Structural racism and adverse maternal health outcomes: a systematic review. Health Place. 2022;78:102923. 10.1016/j.healthplace.2022.102923. 36401939 10.1016/j.healthplace.2022.102923 PMC11216026

[14] “Circumstances contributing to pregnancy-related deaths: data from maternal mortality review committees in 38 U.S. states, 2020.” 2024. [Online]. Available: https://www.cdc.gov/maternal-mortality/php/report/index.html. Accessed 06 Apr 2025.

[15] Hill L, Rao A, Artiga S, Ranji U. “Racial disparities in maternal and infant health: current status and efforts to address them.” 2024. [Online]. Available: https://www.kff.org/racial-equity-and-health-policy/issue-brief/racial-disparities-in-maternal-and-infant-health-current-status-and-efforts-to-address-them/#:~:text=A%20recent%20report%20determined%20that,medication%20they%20thought%20they%20needed. Accessed 06 Apr 2025.

[16] Hung P, et al. Analysis of residential segregation and racial and ethnic disparities in severe maternal morbidity before and during the COVID-19 pandemic. JAMA Netw Open. 2022;5(10):e2237711. 10.1001/jamanetworkopen.2022.37711. 36264572 10.1001/jamanetworkopen.2022.37711 PMC9585430

[17] Ruckart PZ, Ettinger AS, Hanna-Attisha M, Jones N, Davis SI, Breysse PN. The Flint water crisis: a coordinated public health emergency response and recovery initiative. J Public Health Manag Pract. 2019;25(1):S84–90. 10.1097/PHH.0000000000000871. 30507775 10.1097/PHH.0000000000000871 PMC6309965

[18] Wang R, Xi C, Xun L. Something in the pipe: the Flint water crisis and health at birth. J Popul Econ. 2022;35(4):1723–49.

[19] Condello G, et al. Physical activity and health perception in aging: do body mass and satisfaction matter? A three-path mediated link. PLoS ONE. 2016;11(9):e0160805. 10.1371/journal.pone.0160805. 27611689 10.1371/journal.pone.0160805 PMC5017576

[20] Lee S-M, So W-Y, Youn H-S. Importance-performance analysis of health perception among Korean adolescents during the COVID-19 pandemic. Int J Environ Res Public Health. 2021;18(3):1280. 10.3390/ijerph18031280. 33572665 10.3390/ijerph18031280 PMC7908255

[21] Williams ANT, VanArsdale A, Hirschey R, Askelson N, Nash SH. Experiences of racism in health care and medical mistrust shape cancer prevention and control behaviors among Black residents of Black Hawk County, Iowa: a qualitative study. J Racial Ethn Health Disparities. 2024. 10.1007/s40615-024-02199-3. 39379789 10.1007/s40615-024-02199-3 PMC11975716

[22] Henderson KL, Shortridge A, Sadler RC. Environmental crisis or an act of contemporary racism? A flint effect on maternal health disparities. Human Geogr. 2024;17(2):163–77. 10.1177/19427786231199241.

[23] Filippi V, Chou D, Barreix M, Say L. A new conceptual framework for maternal morbidity. Int J Gynecol Obstet. 2018;141:4–9. 10.1002/ijgo.12463.

[24] Ford CL, Airhihenbuwa CO. The public health critical race methodology: praxis for antiracism research. Soc Sci Med. 2010;71(8):1390–8. 10.1016/j.socscimed.2010.07.030. 20822840 10.1016/j.socscimed.2010.07.030

[25] Howell EA. Reducing disparities in severe maternal morbidity and mortality. Clin Obstet Gynecol. 2018;61(2):387–99. 10.1097/GRF.0000000000000349. 29346121 10.1097/GRF.0000000000000349 PMC5915910

[26] Mendelsohn AL, et al. Reading aloud, play, and social-emotional development. Pediatrics. 2018;141(5):e20173393. 10.1542/peds.2017-3393. 29632254 10.1542/peds.2017-3393 PMC5914489

[27] Centers for Disease Control and Prevention. “Are PRAMS data available to researchers?” Centers for Disease Control and Prevention. https://www.cdc.gov/prams/php/data-research/index.html

[28] “Severe maternal morbidity in the United States,” Centers for Disease Control and Prevention. https://www.cdc.gov/maternal-infant-health/php/severe-maternal-morbidity/icd.html

[29] Rice F, et al. Agreement between maternal report and antenatal records for a range of pre and peri-natal factors: the influence of maternal and child characteristics. Early Hum Dev. 2007;83(8):497–504. 10.1016/j.earlhumdev.2006.09.015. 17071023 10.1016/j.earlhumdev.2006.09.015

[30] “RStudio: integrated development for R. Rstudio.” PBC, Boston, MA; 2020. [Online]. Available: http://www.rstudio.com/. Accessed 30 Aug 2024.

[31] Kahle D, Wickham H. “ggmap: spatial visualization with ggplot2.” R J 2013;5(1). [Online]. Available: https://journal.r-project.org/archive/2013-1/kahle-wickham.pdf. Accessed 13 Nov 2024.

[32] Ruggles S, Flood S, Goeken R, Schouweiler M, Sobek M. “IPUMS USA: Version 12.0 .” Minneapolis, MN: IPUMS; 2022.

[33] “QGIS Geographic Information System.” QGIS.org. [Online]. Available: http://www.qgis.org. Accessed 13 Nov 2024.

[34] Petersen EE, et al. Racial/ethnic disparities in pregnancy-related deaths — United States, 2007–2016. MMWR Morb Mortal Wkly Rep. 2019;68(35):762–5. 10.15585/mmwr.mm6835a3. 31487273 10.15585/mmwr.mm6835a3 PMC6730892

[35] Kumari U, Sharma RK, Keshari JR, Sinha A. Environmental exposure: effect on maternal morbidity and mortality and neonatal health. Cureus. 2023;15(5):e38548. 10.7759/cureus.38548. 37273345 10.7759/cureus.38548 PMC10239284

[36] Howell EA, Egorova NN, Balbierz A, Zeitlin J, Hebert PL. Site of delivery contribution to black-white severe maternal morbidity disparity. Am J Obstet Gynecol. 2016;215(2):143–52. 10.1016/j.ajog.2016.05.007. 27179441 10.1016/j.ajog.2016.05.007 PMC4967380

[37] Mujahid MS, et al. Birth hospital and racial and ethnic differences in severe maternal morbidity in the state of California. Am J Obstet Gynecol. 2021;224(2):219.e1-219.e15. 10.1016/j.ajog.2020.08.017. 32798461 10.1016/j.ajog.2020.08.017 PMC7855283

[38] Greenwood BN, Hardeman RR, Huang L, Sojourner A. Physician–patient racial concordance and disparities in birthing mortality for newborns. Proc Natl Acad Sci. 2020;117(35):21194–200. 10.1073/pnas.1913405117. 32817561 10.1073/pnas.1913405117 PMC7474610

[39] Serafini K, et al. Racism as experienced by physicians of color in the health care setting. Fam Med. 2020;52(4):282–7. 10.22454/FamMed.2020.384384. 32267524 10.22454/FamMed.2020.384384

[40] Kruger DJ, French-Turner T, Brownlee S. Genesee County REACH Windshield Tours: enhancing health professionals understanding of community conditions that influence infant mortality. J Prim Prev. 2013;34(3):163–72. 10.1007/s10935-013-0301-8. 23605377 10.1007/s10935-013-0301-8

[41] Baumeister H, Kriston L, Bengel J, Härter M. High agreement of self-report and physician-diagnosed somatic conditions yields limited bias in examining mental–physical comorbidity. J Clin Epidemiol. 2010;63(5):558–65. 10.1016/j.jclinepi.2009.08.009. 19959329 10.1016/j.jclinepi.2009.08.009

[42] Furuta M, Sandall J, Bick D. A systematic review of the relationship between severe maternal morbidity and post-traumatic stress disorder. BMC Pregnancy Childbirth. 2012;12(1):125. 10.1186/1471-2393-12-125. 23140343 10.1186/1471-2393-12-125 PMC3582425

[43] Prinja S, Jeet G, Kumar R. Validity of self-reported morbidity. Indian J Med Res. 2012;136(5):722–4. 23287117 PMC3573591

